# Synergistic cardiac pathological hypertrophy induced by high-salt diet in IGF-IIRα cardiac-specific transgenic rats

**DOI:** 10.1371/journal.pone.0216285

**Published:** 2019-06-18

**Authors:** Ruey-Lin Chang, Srinivasan Nithiyanantham, Chih-Yang Huang, Pei-Ying Pai, Tung-Ti Chang, Lai-Chin Hu, Ray-Jade Chen, V. VijayaPadma, Wei-Wen Kuo, Chih-Yang Huang

**Affiliations:** 1 School of Post-Baccalaureate Chinese Medicine, College of Chinese Medicine, China Medical University, Taichung, Taiwan; 2 Graduate Institute of Basic Medical Science, China Medical University, Taichung, Taiwan; 3 Translation Research Core, China Medical University Hospital, China Medical University, Taichung, Taiwan; 4 Division of Cardiology, China Medical University Hospital, Taichung, Taiwan; 5 School of Chinese Medicine, China Medical University, Taichung, Taiwan; 6 Department of Internal Medicine, Division of Cardiology, Armed Forces Taichung General Hospital, Taichung, Taiwan; 7 Department of Surgery, School of Medicine, College of Medicine, Taipei Medical University, Taipei, Taiwan; 8 Department of Biotechnology, Bharathiar University, Coimbatore, India; 9 Department of Biological Science and Technology, China Medical University, Taichung, Taiwan; 10 College of Medicine, Hualien Tzu Chi Hospital, Buddhist Tzu Chi Medical Foundation, Tzu Chi University, Hualien, Taiwan; 11 Department of Medical Research, China Medical University Hospital, China Medical University, Taichung, Taiwan; 12 Department of Biotechnology, Asia University, Taichung, Taiwan; Universidade Federal do Rio de Janeiro, BRAZIL

## Abstract

Stress-induced cardiac hypertrophy leads to heart failure. Our previous studies demonstrate that insulin-like growth factor—II receptor (IGF-IIR) signaling is pivotal to hypertrophy regulation. In this study, we show a novel IGF-IIR alternative spliced transcript, IGF-IIRα (150 kDa) play a key role in high-salt induced hypertrophy mechanisms. Cardiac overexpression of IGF-IIRα and high-salt diet influenced cardiac dysfunction by increasing pathophysiological changes with up-regulation of hypertrophy markers, atrial natriuretic peptide (ANP) and brain natriuretic peptide (BNP). We found that, cardiac hypertrophy under high-salt conditions were amplified in the presence of IGF-IIRα overexpression. Importantly, high-salt induced angiotensin II type I receptor (AT1R) up regulation mediated IGF-IIR expressions via upstream mitogen activated protein kinase (MAPK)/silent mating type information regulation 2 homolog 1 (SIRT1)/heat shock factor 1 (HSF1) pathway. Further, G-coupled receptors (Gαq) activated calcineurin/nuclear factor of activated T-cells, cytoplasmic 3 (NFATc3)/protein kinase C (PKC) signaling was significantly up regulated under high-salt conditions. All these effects were observed to be dramatically over-regulated in IGF-IIRα transgenic rats fed with a high-salt diet. Altogether, from the findings, we demonstrate that IGF-IIRα plays a crucial role during high-salt conditions leading to synergistic cardiac hypertrophy.

## Introduction

Insulin-like growth factor (IGF) and IGF-II receptor (IGF-IIR) signaling is crucial for cardiac development and remodelling [1–3]. IGF-IIR is parentally imprinted and knocking down its expression had severe fetal cardiac abnormalities [4,5]. Reactivation of IGF-IIR signaling occurs during cardiac stresses leading to cardiac remodeling; thus prolonged stress ensues with cardiac hypertrophy and heart failure. IGF-IIR, a type I transmembrane glycoprotein activation and its cell surface expression in cardiomyocytes promote IGF-II binding through G-protein-related mechanism leading to cardiomyocyte apoptosis [3,6]. Substantial evidence from our laboratory demonstrates that IGF-II:IGF-IIR signaling promotes physiological and pathological changes in the heart tissue leading to cardiac hypertrophy, apoptosis and heart failure [3,6–8]. We have made pioneering studies in identifying the molecular pathway of IGF-IIR signaling; we elucidated IGF-IIR activation in angiotensin II (ANG II)-induced hypertensive cardiomyocyte apoptosis through JNK activated SIRT1 degradation leading to HSF1 acetylation [3]. We identified CHIP mediated HSF1 protein stability via its TPR domain is essential for HSF1 nuclear translocation and subsequent inhibition of IGF-IIR expression [9]. In addition, we also found that ERK/GSK3 mediated HSF1 phosphorylation and subsequent RNF126 degradation by ANG II caused IGF-IIR protein stabilization leading to hypertrophy [10]. Thus, these studies showed the clear evidence that IGF-IIR activation and its overexpression is responsible for cardiac hypertrophy and heart failure. Importantly, in IGF-IIR knockdown studies, we did not find complete recovery from DOX-induced cardiomyocyte apoptosis [9]. Thus, implicating on the association of other key regulatory proteins in cardiac hypertrophy mechanisms.

Recently, we identified novel alternative splicing truncated IGF-IIR using rapid amplification of cDNA ends (RACE) and sequence analysis. This fragment lacked IGF-IIR exon 1–9 segment but consisted of intron 9 (nt 645–806)- exon10- intron 36 (nt 1–455). mRNA expression pattern for primer specific to intron 9 (nt 645–806) revealed its expression in heart, brain, liver, placenta and testis of rats. Further, we also confirmed that this transcript can encode a protein with 1359 amino acids with start codon at 231 bp (exon 10) and stop codon at 4307 bp (intron 36). By sequence analysis, we found that amino acids of the truncated protein were consistent with IGF-IIR, except the C-terminal 15 amino acid. We named the novel protein as IGF-IIRα and aimed to identify its biological significance and its involvement in cardiac pathophysiology.

IGF-IIRα regulates cardiac apoptosis through down-regulation of survival proteins AKT/PI3K signaling and up-regulation of caspase 3 activation. In addition, overexpression of IGF-IIRα regulates cardiac fibrosis through uPA/tPA/TGF-β signaling and higher collagen accumulation and further aggravated its effect in high-salt condition [11]. In this study, we aimed to identify whether novel IGF-IIRα is involved in cardiac hypertrophy and further its functional role in high-salt induced hypertensive heart failure *in vivo*. Then, we would like to investigate whether IGF-IIRα could be a novel potential therapeutic target for heart failure.

## Materials and methods

### Antibodies and reagents

All chemicals and reagents were procured from Sigma-Aldrich, USA. For western blotting, the primary antibodies p-ERK, p-JNK, p-P38 were purchased from Cell Signalling, USA. IGF-IIR, AT1R, NFATC3, ANP, BNP, α-tubulin, p-PKC (Abcam, USA) and GAPDH, SIRT1, Gαq, p-GATA4 (Santa Cruz Biotechnology, USA). All secondary antibodies (anti-rabbit, mouse and goat, HRP-conjugated antibodies) were procured from Santa Cruz Biotechnology, USA.

### Transgenic construction

The transgenic construct includes the rMYH6 promoter, 3HA-IGF-IIRα-A2 (NCBI) BAC DNA. 3HA-IGF-IIRα-A2 was amplified and ligated into the rMYH6 promoter expression vector. The primer sequences used include IGF-IIRα:5’IGF-IIRα-KnpI:5’-TTGGTACCGAATGAGTGTCATAAACTTTGAG-3’and3’-IGF-IIRα-XbaI:5’-CCCTCTAGAAGTCATGTCGGCTGCTGTGAGTGAAGCTGA-3’. We next successfully subcloned the full-length of IGF-IIRα into the pcDNA3.1-myc-His driven by α-MHC cardiac-specific promoter via pronuclear microinjection and developed IGF-IIRα over expression transgenic rats (TG). Positive founders were identified by PCR and backcrossed to WT. Genotyping was performed by PCR analyses with the specific primers. Forward primer5’-TAGCAAACTTCAGCCACCCTTC-3’ and Reverse primer 5’-ACTTCCACTCTTATCCACAGCACAC-3’ which were designed to amplify a 739bp fragment.

### Animal procedure

All protocols were reviewed and approved by the IRB (Institutional Review Board) and the animal care and use advisory group of the China Medical University, Taichung, Taiwan. Animals were procured from BioLasco Co., Ltd., Taipei, Taiwan. Male TG founder had a deficiency in fertility. In our study, we have used eight week oldfemale Sprague-Dawley (SD) animals were supplied with standard diet (Laboratory rodent diet 5001) & tap water and maintained at a constant temperature (22°C) on a 12-hour light/dark cycle. After a 4 week acclimatisation period, the animals were divided into 4 groups with 6 animals in each group: SD rats (WT), SD-TG (IGF-IIRα) rats (TG), SD + high salt diet rats (8% high salt) (WT-HD), SD-TG (IGF-IIRα) + high salt diet rats (8% high salt) (TG-HD). The treatment period is for about 16 weeks. The high salt diets are procured from research diets, NJ, USA. After treatment, all rats were sacrificed by decapitation under terminal anesthesia and hearts were collected. Finally, the heart tissue was collected and stored at -80°C for further analysis.

### Echocardiography

Echocardiography was performed for SD rats, SD-TG (IGF-IIRα) rats, SD + high salt diet rats (8% high salt), SD-TG (IGF-IIRα) + high salt diet rats (8% high salt) before sacrificing. Rats were anesthetized with isoflurane, and echocardiography was performed using 12 MHz linear transducers and 5–8 MHz sector transducer (Vivid 3, General Electric Medical Systems Ultrasound, Tirat Carmel, Israel). Measurements were contrived from M-mode planes and two dimensional images obtained in the parasternal long and short axesat the level of the papillary muscles after observation of at least six cardiac cycles. IVSd—Interventricular septal thickness at end-diastole, LVIDd—Left ventricular internal dimension at end-diastole, LVPWd—Left ventricular posterior wall thickness at end-diastole, IVSs—Interventricular septal thickness at end-systole, LVIDs—Left ventricular internal dimension at end-systole, LVPWs—Left ventricular posterior wall thickness at end-systole, SV–Stroke Volume, LVd Mass—Left ventricular end diastole mass, LVs Mass–Left ventricular end systole mass were measured.

### Tissue protein extraction

The left ventricle tissue was collected and homogenized using lysis buffer (20mM Tris, 2mM EDTA, 50mM β-mercaptoethanol, 10% glycerol, protease inhibitor, phosphatase inhibitor, pH 7.4). The homogenates were kept at -20°C for overnight and centrifuged at 12,000 rpm for 30 minutes. After centrifugation, the supernatant was collected and stored at -80°C for further analysis.

### Western blotting

The western blotting for protein expression analysis was as described previously with slight modifications [12]. The Protein concentration of the heart tissue was estimated by Lowry's protein assay. Protein samples 40 μg/lane were resolved by 8–15% gradient SDS-PAGE with a constant voltage. Then the gel was transferred to a PVDF membrane (GE Healthcare Life Sciences) for 90 minutes at 90V. After transferring, the membrane was placed in blocking solution (3% BSA) for one hour at RT. After washing with TBST, the membrane was incubated with respective primary antibody overnight at 4°C. Then, the membrane was incubated with secondary antibody for one hour at RT. Then the blots were visualized using a chemiluminescence ECL western blotting reagent (Millipore) in Fujifilm LAS-3000 (GE Healthcare). The intensities were quantified using ImageJ.

### Hematoxylin and eosin staining

The heart was fixed in 4% buffered formaldehyde and 2-μm thick sections were cut from paraffin-embedded tissue blocks. For histopathological staining, the slices were counterstained with hematoxylin-eosin following manufacturer's instructions. The average diameter of cardiomyocyte was analyzed with image analysis software. All measurements were averaged from three slices.

### Statistical analysis

Statistical analysis was performed with GraphPad Prism software, version 6.01, California. All data are expressed as Mean± SD. The data were subjected to two-way analysis of variance followed by Tukey's post hoc tests. The level of statistical significance and confidence level was set as p<0.05.

## Results

### IGF-IIRα transgenic rat development

**Fig 1:**
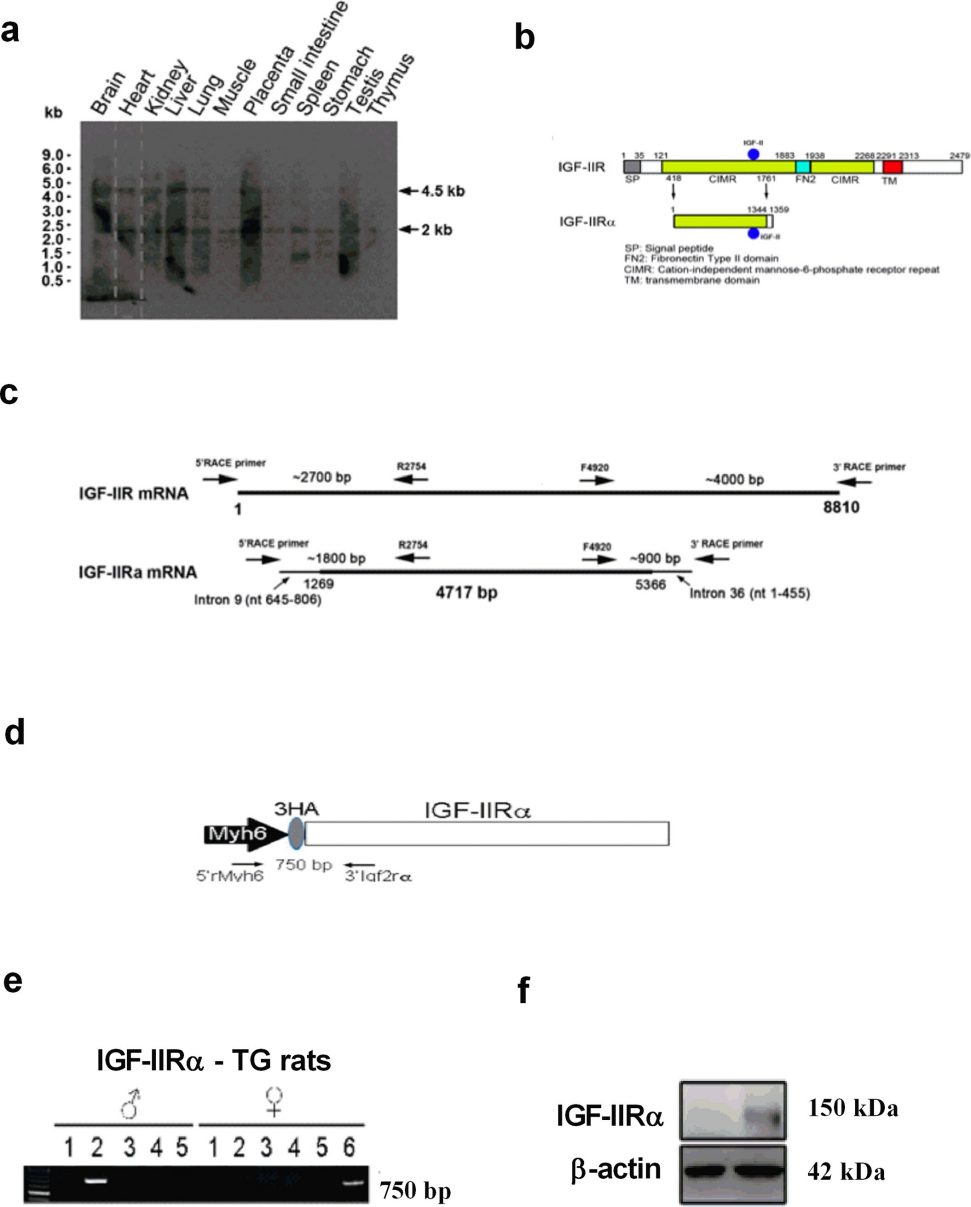
Identification of alternative splicing form of IGF-IIR: IGF-IIRα and development of IGF-IIRα transgenic rats. (a) identification of novel IGF-IIRα mRNA transcripts contained intron9 (nt 645–806). Detection of mRNA transcripts by northern blots using random primed DNA labeling probe contained intron9 (nt 645–806) in various tissue samples, (b) the structural domain of IGF-IIR and IGF-IIRα, (c) full length of IGF-IIR and IGF-IIRα mRNA, (d) schematic diagram of IGF-IIRα transgenic vector construction. IGF-IIRα with N-terminal 3XHA, which is driven by cardiac specific promoter Myh6 promoter, (e) DNA genotyping was performed by PCR for TG-IGF-IIRα positive founders identification and (f) protein expression of TG-IGF-IIRα from the heart.

Fig 1A shows IGF-IIR and novel IGF-IIRα mRNA transcripts by northern blot using random primed DNA labelling probe contained intron 9 in different organs of rat. Fig 1B shows the structural domain of IGF-IIR and IGF-IIRα. IGF-IIRα is identified as the truncated form of IGF-IIR having CIMR (cation-independent mannose-6-phosphate receptor repeat) region. Full length of IGF-IIR and IGF-IIRα mRNA region is shown in the Fig 1C. Schematic diagram of IGF-IIRα transgenic vector construction with N-terminal 3XHA, the vector was driven by cardiac specific promoter Myh6 promoter (Fig 1D). Two transgenic founders of IGF-IIRα rats (one male and one female) were identified and offspring (F1) TG-IGF-IIRα were developed. The genomic DNA amplification of IGF-IIRα was shown in Fig 1E. Further, the protein expression of IGF-IIRα was confirmed in the TG-IGF-IIRα rat heart (Fig 1F).

### IGF-IIRα induced cardiac damage in normal and high-salt induced diet

**Fig 2:**
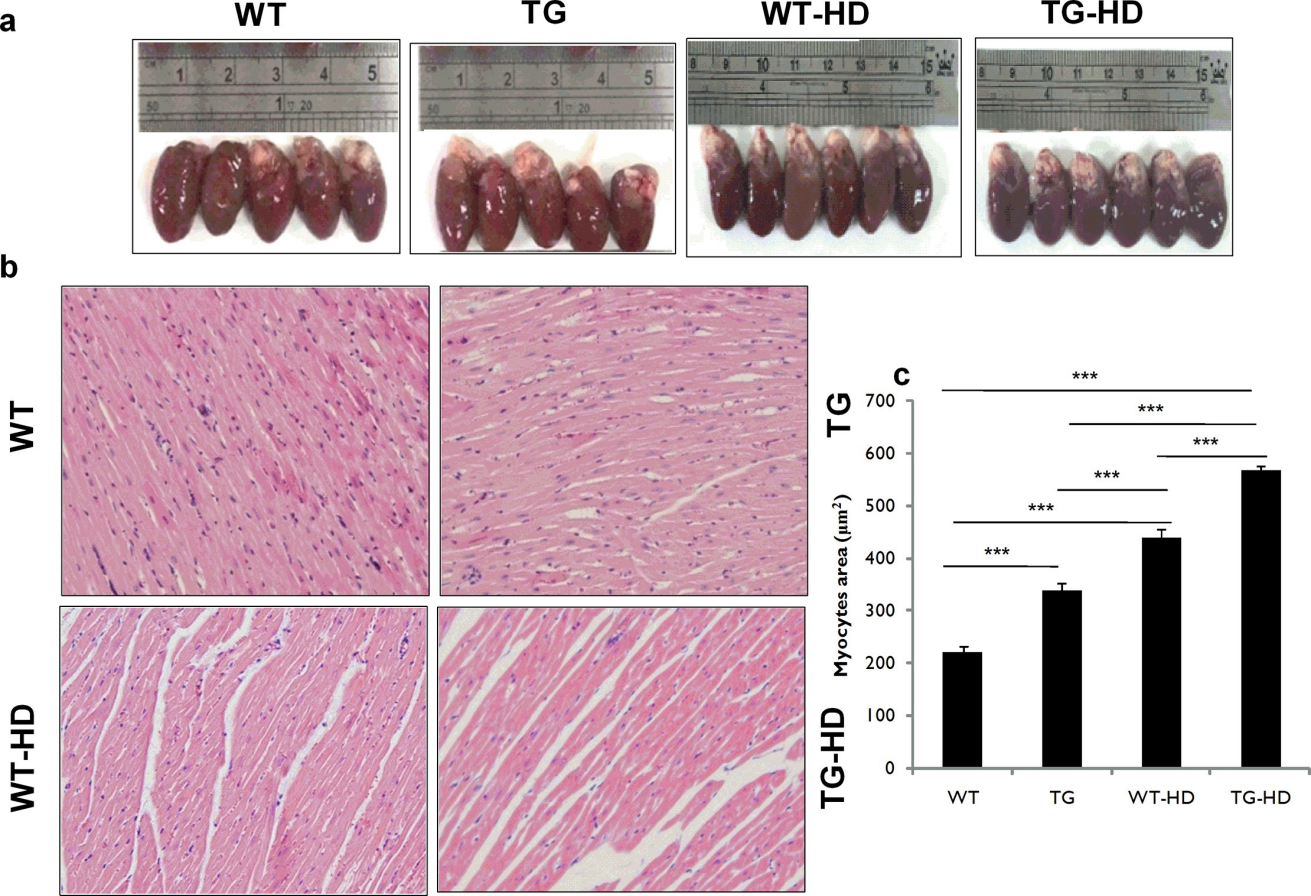
Structural and morphological changes in heart tissues of TG-IGF-IIRα rats. (a) heart of TG-IGF-IIRα rats dramatically enlarged and (b,c) representative histological images with hematoxylin and eosin staining of heart sections. Histopathological analysis reveals that high-salt induced hypertrophy and severe heart damages in TG-IGF-IIRα rats.***: p<0.001.

We identified that the TG-HD and WT-HD rats showed an increase in their heart size compared to WT rats. TG-HD showed heart enlargement compared to its counterpart TG rats (Fig 2A). The histologic cardiomyocytes area was significantly higher in TG-HD, WT-HD, TG compared to the WT (Fig 2B and 2C). Morphological data are summarized in Table 1. There was no significant difference in body weight and tibia among the groups. There was a significant difference in WHW, WHW/tibia, WHW/BW between the groups. In the present study, we observed that whole heart weight increases in WT-HD and TG-HD rats compared to WT rats. These results suggest that IGF-IIRα regulates cardiac hypertrophy.

**Table 1:** Body weight, cardiac characteristics and echocardiographic parameters of the normal diet and high salt diet groups.

	WT	TG	WT-HD	TG-HD
BW (g)	262.25 ± 21.06	265.4 ± 26.03	268 ± 18.91	274 ± 17.82
WHW (g)	0.77 ± 0.05	0.82 ± 0.03	1.07 ± 0.06 ^c^^,^ ^f^	1.12 ± 0.08 ^c^^,^ ^f^
Tibia (mm)	39.5 ± 2.38	40.60 ± 2.07	40.48 ± 0.79	40.02 ± 0.19
WHW/Tibia (100g/mm)	1.95 ± 0.20	2.02 ± 0.14	2.64 ± 0.14 ^c^^,^ ^f^	2.79 ± 0.19 ^c^^,^ ^f^
WHW/BW × 10^3^	2.93 ± 0.09	3.10 ± 0.26	4.00 ± 0.37 ^c^^,^ ^f^	4.25 ± 0.06 ^c^^,^ ^f^
IVSd (mm)	0.97 ± 0.07	1.16 ± 0.01 ^c^	1.13 ± 0.03 ^c^	1.21 ± 0.06 ^c^^,^ ^g^
LVIDd (mm)	7.92 ± 0.13	8.58 ± 0.10 ^c^	8.10 ± 0.32^e^	9.25 ± 0.22 ^c^^,^ ^f^^,^ ^i^
LVPWd (mm)	0.92 ± 0.06	0.96 ± 0.07	1.12 ± 0.07 ^b^^,^ ^e^	1.35 ± 0.10 ^c^^,^ ^f^^,^ ^i^
IVSs (mm)	1.83 ± 0.16	2.14 ± 0.03^a^	2.04 ± 0.06	2.82 ± 0.31 ^c^^,^ ^f^^,^ ^i^
LVIDs (mm)	4.51 ± 0.29	5.43 ± 0.05 ^c^	4.60 ± 0.13^e^	5.63 ± 0.60 ^c^^,^ ^i^
LVPWs (mm)	1.57 ± 0.06	1.91 ± 0.06^b^	1.99 ± 0.02 ^c^	2.46 ± 0.29 ^c^^,^ ^f^^,^ ^i^
SV (Teich)	1.29 ± 0.07	0.98 ± 0.03 ^c^	0.92 ± 0.08 ^c^	0.91 ± 0.06 ^c^
LVd Mass (ASE)	1.02 ± 0.01	1.10 ± 0.08	1.10 ± 0.05	1.21 ± 0.03 ^c^^,^ ^e^^,^ ^h^
LVs Mass (ASE)	1.04 ± 0.03	1.19 ± 0.02 ^c^	1.18 ± 0.04 ^c^	1.30 ± 0.05 ^c^^,^ ^f^^,^ ^i^

Values are Mean ± SD.

^a^ p<0.05

^b^ p<0.01 and

^c^ p<0.001 are compared to wild type normal diet

^d^ p<0.05

^e^ p<0.01 and

^f^ p<0.001 are compared to TG normal diet

^g^ p<0.05

^h^ p<0.01 and

^i^ p<0.001 are compared to wild type high salt diet. BW—Body weight; WHW—Whole heart weight; IVSd—Interventricular septal thickness at end-diastole; LVIDd—Left ventricular internal dimension at end-diastole; LVPWd—Left ventricular posterior wall thickness at end-diastole; IVSs—Interventricular septal thickness at end-systole; LVIDs—Left ventricular internal dimension at end-systole; LVPWs—Left ventricular posterior wall thickness at end-systole; SV–Stroke Volume; LVd Mass—Left ventricular end diastole mass; LVs Mass–Left ventricular end systole mass.

The echocardiography parameters of IGF-IIRα in the normal diet and high salt diet data are presented in Table 1. There was a significant difference between WT & TG subsequently differences are found in WT-HD & TG-HD. Echocardiography parameters like IVSd, LVIDd, LVPWd, IVSs, LVIDs, LVPWs, SV, LVd Mass and LVs Mass were highly up-regulated in TG-HD compared to WT. Consequently, there was a significant difference between WT and TG—i.e IVSd, LVIDd, LVIDs, SV and LVs Mass. These observations showed that, IGF-IIRα is a key player in cardiac damage; while supplementation with high-salt diet further aggravated IGF-IIRα mediated heart damage.

### IGF-IIRα is responsible for cardiac hypertrophy

**Fig 3:**
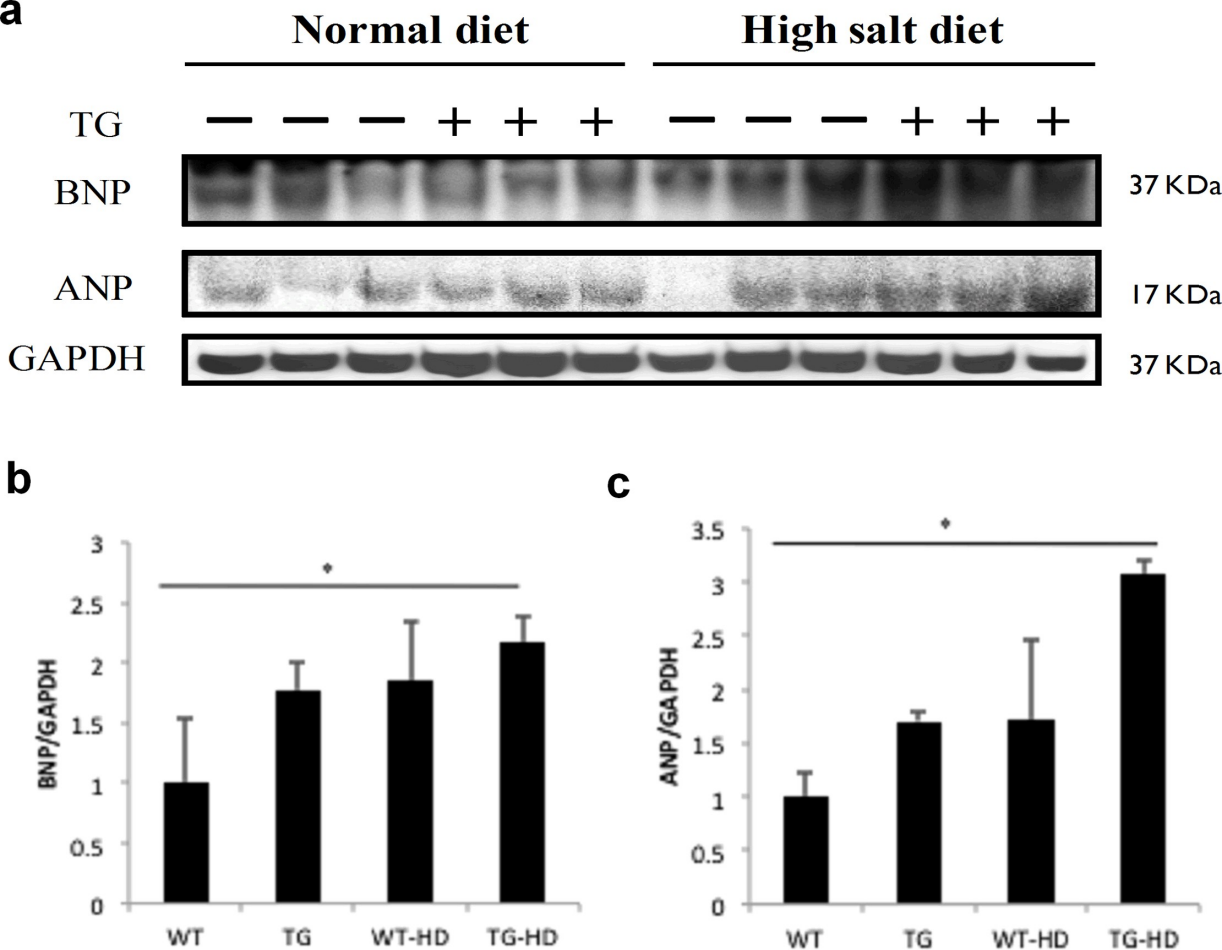
IGF-IIRα regulates cardiac hypertrophy. (a) protein levels of hypertrophy markers BNP and ANP, (b) quantified expression levels of BNP marker expression in WT, TG, WT-HD & TG-HD and (c) cardiac hypertrophy marker ANP expression in WT, TG, WT-HD & TG-HD. Representative western blots are shown *: p<0.05.

Our previous studies demonstrate that IGF-IIR regulates cardiac hypertrophy mechanism [10]. So, in this study, we aimed to understand whether IGF-IIRα, the alternative splicing form of IGF-IIR might involve in the regulation of hypertrophy. Further, its role in high-salt fed rats will show its functional importance during stress conditions. In our present study, we found that WT-HD and TG-HD rats showed increased ANP & BNP hypertrophy marker expressions compared to WT rats (Fig 3). Further, ANP and BNP expressions were significantly higher in TG-HD compared to WT rats. These findings showed that IGF-IIRα regulates cardiac hypertrophy and further causes extensive activation of hypertrophy markers during high-salt conditions.

### IGF-IIRα regulates upstream MAP Kinase activation

**Fig 4:**
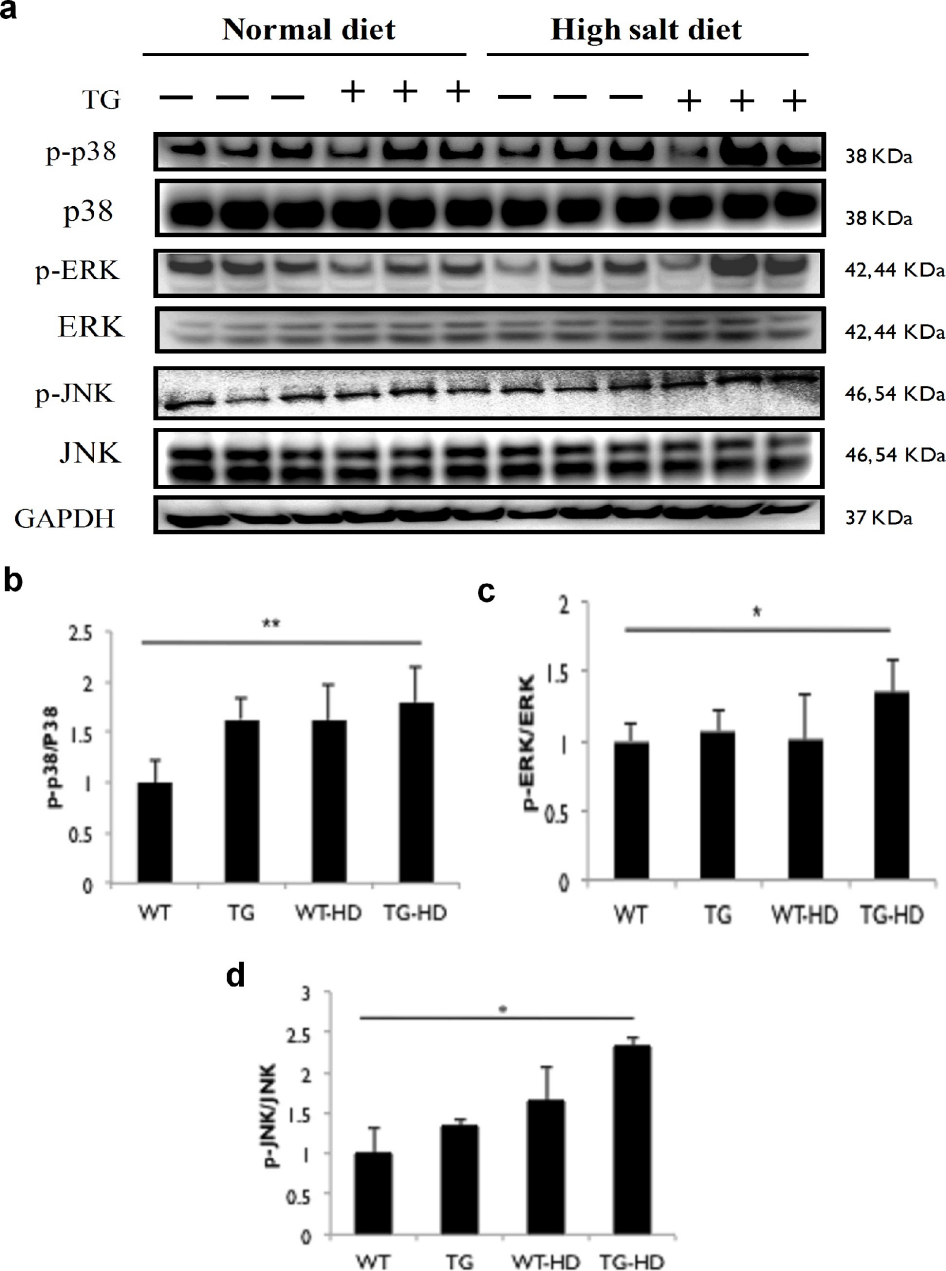
Overexpression of IGF-IIRα up-regulates MAP kinase pathway in high salt diet groups. (a) protein expression of hypertrophy related MAPK protein levels in WT, TG, WT-HD & TG-HD and (b-d) bars represent the relative protein quantification and indicates Mean ± SD. Representative western blots are shown *: p<0.05, **: p<0.01.

Our previous findings reported that IGF-IIR:IGF-II signaling activates p-JNK expression in angiotensin induced cardiomyocyte apoptosis [3]. In this study, we found that IGF-IIRα TG rats showed up regulated p-ERK, p-JNK and p-p38 expressions compared to WT rats (Fig 4). We found significant up-regulation of p-p38 expressions in TG-HD compared to WT rats. The p-JNK and p-ERK expressions in TG-HD rats were significantly higher compared to WT rats. We observed that TG rats by itself increased MAPK expressions and these expressions were further up-regulated in high salt conditions. In general, these findings showed that IGF-IIRα regulates hypertrophy and hyperactivation occurs during salt supplementation.

### IGF-IIRα regulates IGF-IIR signaling through SIRT1 degradation and HSF1 expression

**Fig 5:**
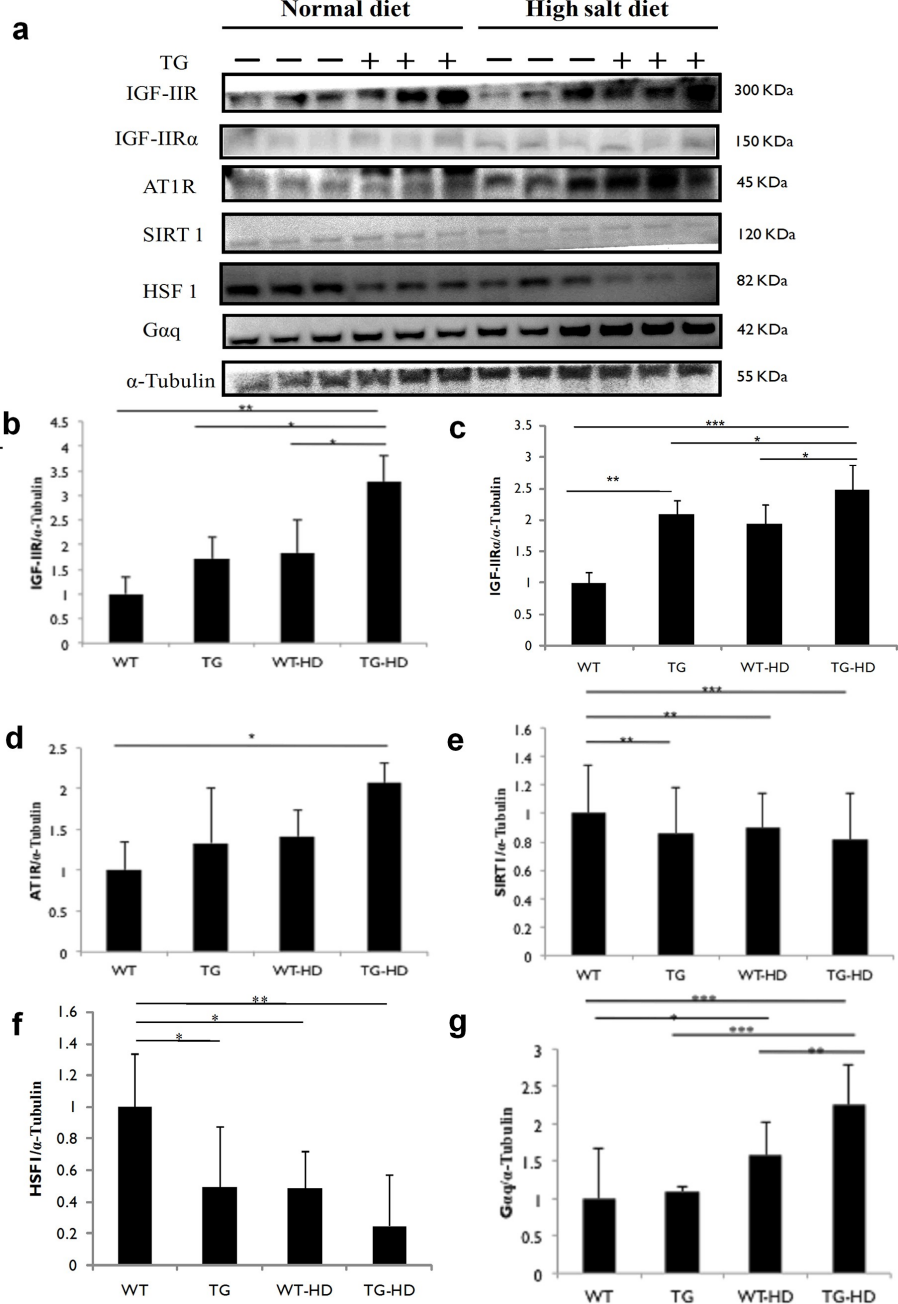
IGF-IIRα regulates IGF-IIR signaling through SIRT1 degradation and HSF1 acetylation. (a) expression levels of IGF-IIR, IGF-IIRα, AT1R, SIRT1, HSF1 and Gαq. Protein expression of cardiac tissue (b) IGF-IIR,(c) IGF-IIRα,(d) AT1R,(e) SIRT1, (f) HSF1 and (g) Gαq. Representative western blots are shown *: p<0.05, **: p<0.01, ***: p<0.001.

We next sort to examine the mechanism of IGF-IIRα regulated cardiac hypertrophy. We found that there was a significant increase in IGF-IIR and IGF-IIRα expressions in TG-HD rats compared to TG rats. Further, TG-HD rats showed an increase in AT1R expressions compared to TG rats (Fig 5). Downstream to this, we found there was an increased SIRT1 degradation with concomitant HSF1 down regulation during high salt supplementation. HSF1 protein expression was significantly down-regulated in TG-HD compared to WT rats, TG rats and WT-HD rats. Significant up-regulation of Gαq expressions was observed in TG-HD rats as compared to TG and WT rats. These results showed that IGF-IIRα regulates hypertrophy via activation of SIRT1 degradation and HSF1 acetylation.

### IGF-IIRα regulates pathological hypertrophy through Calcineurin/NFATc3 pathway

**Fig 6:**
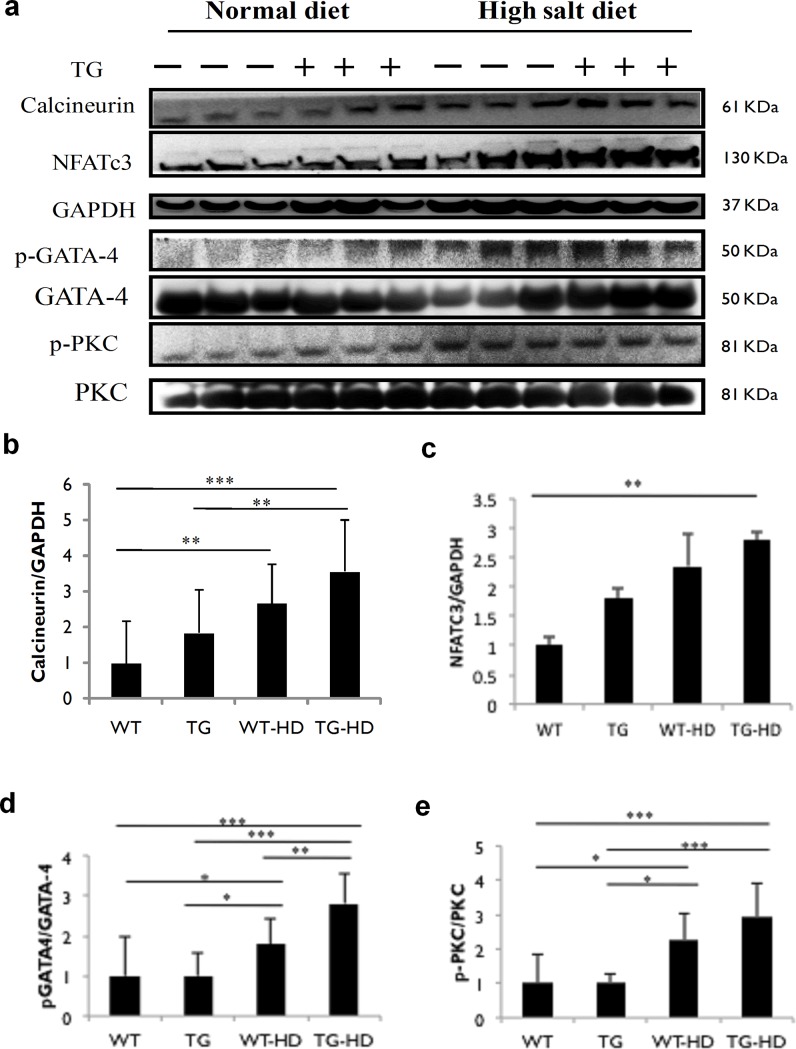
TG-IGF-IIRα causes pathological hypertrophy in high-salt diet groups. (a) expression levels of calcineurin, GATA-4, nuclear localization of NFATc-3 and PKC. Protein expression of cardiac tissue (b) Calcineurin, (c) NFATc3, (d) pGATA-4 and (e) pPKC. Representative western blots are shown *: p<0.05, **: p<0.01, ***: p<0.001.

Gαq mediated calcineurin/NFATc3 intracellular signaling leads to ANP and BNP expressions. In the present findings, the results showed up-regulated expressions of calcineurin and NFATc3 in TG and high salt diet rats (Fig 6). There was a significant up-regulation of calcineurin expression in TG-HD rats compared to TG rats and WT rats. Calcineurin-induced transcription factor NFATc3 expression was found to be statistically significant in TG-HD rats compared to WT rats. Further, p-GATA4 expressions were up-regulated in TG-HD rats as compared with TG and WT rats. Additionally, p-PKC expression was up-regulated in TG-HD rats compared to TG and WT rats. From the above results, it is evident that activation of calcineurin/NFATc3 signaling by IGF-IIRα regulates pathological hypertrophy.

## Discussion

We demonstrated for the first time that, IGF-IIRα plays a key regulatory role in the cardiac structure and high-salt induced hypertrophy. In this study, we identified IGF-IIRα overexpression aggravated stress-induced cardiac dysfunction by enhancing up-stream AT1R/HSF1 pathway and Gαq-mediated calcineurin/NFATc3 signaling leading to pathological hypertrophy.

Remarkable findings from our lab shows that IGF-IIR signaling regulates cardiac hypertrophy leading to heart failure [10]. Here, we report on the association of novel IGF-IIRα in the regulation of hypertrophy. In this study, we demonstrate the association of IGF-IIRα and high-salt induced hypertension in regulating cardiac hypertrophy. Adaptive response to high-salt induced hypertension initiates physiological changes to the heart which then transmits to maladaptive cardiac dysfunction leading to pathological hypertrophy [13,14]. The present observations on the high-salt induced histological changes and increased cardiac LV mass were consistent with the previous reports [15–17]. Data from the morphological and pathological findings revealed that, IGF-IIRα overexpression is involved in cardiac remodeling and cardiac function decline. IGF-IIRα overexpressed rats showed visible changes in heart weight, heart weight-body weight ratio, a cross-sectional area of the heart. As LVIDd and LVIDs are recognized index for cardiac hypertrophy progress, significantly higher IVSs, IVSd, LVPWd, LVIDd and LV mass values indicated left ventricular abnormality in IGF-IIRα rats. In our present study, we assessed the difference in LVd mass and LVs mass in TG, WT-HD and TG-HD compared to the control rats. The present findings results are similar to the Zheng et al. [18] and Crawford et al. [19] reported that differences found between LV mass at end diastole and systole in healthy patients and coronary artery disease/hypertensive patients. There is no significant difference between WT and TG in WHW/BW and a few echocardiographic parameters. Basically, IGF-IIRα gene is in silent form, under stress condition it activates and leads to cardiac remodeling. But in some important parameters we observed a significant difference between WT and TG in IVSd, LVIDd, LVIDs, SV and LVs mass. Chang et al. [11] reported that ejection fraction and fractional shortening was down regulated in TG, WT-HD and TG-HD compared to the WT rats. In addition, IGF-IIRα regulates cardiac apoptosis through cytochrome C/caspase 3 activation with decreased expression of survival proteins and cardiac fibrosis through uPA/tPA/TGF-β signaling and aggravates in high-salt condition. We and others have reported that reactivation of hypertrophy markers ANP and BNP leads to cardiac hypertrophy through Gαq mediated IGF-IIR signaling [10,20–23]. Activated to restore the heart function, these natriuretic peptides regulate vasodilatation and peripheral vascular resistance [24]. However, paralleled enhancement of various signaling pathways leads to vascular dysfunction and matrix remodeling with end result causing heart failure. The observed mild increase in hypertrophy markers in IGF-IIRα overexpression rats reveal that IGF-IIRα might independently regulate hypertrophy mechanisms. These results give us previously unanswered questions about incomplete recovery from doxorubicin mediated cardiac dysfunction during IGF-IIR silencing [9]. Importantly, IGF-IIRα overexpression under high-salt conditions highly amplified cardiac dysfunction indicating its major participation in stress-induced cardiac hypertrophy. In addition to previously demonstrated significant role of IGF-IIR [3,9], the present finding adds on the functional contribution of IGF-IIRα in hypertrophy mechanisms.

IGF-IIR: IGF-II signalling regulates pathological hypertrophy. Earlier we have demonstrated that AT1R mediated JNK1/2 activation leading to HSF1 acetylation through SIRT1 degradation up-regulates IGF-IIR expression [3]. MAPK, a serine threonine kinase regulates stress-induced activation of intra-cellular signaling [25,26]. Here, we observed a significant up-regulation in the phosphorylated status of ERK1/2, JNK as well as p38 in cardiac tissues in TG, WT-HD and TG-HD rats. However, TG-HD rats showed the significant increase in MAPK expressions compared to WT and TG rats. Activation of MAPK is central to cardiomyocyte hypertrophy which is regulated via ANG-II mediated AT1R activation [27]. AT1R is also known to mediate ANG-II-induced physiological changes including vasoconstriction, retention of salt and water and cardiac contractility [28]. Under high-salt conditions, we found significant activation of IGF-IIR signaling through increased HSF1 acetylation via SIRT1 degradation. Interestingly, we found that IGF-IIR expressions in TG rats were accompanied by a slight increase in AT1R, Gαq and decrease in HSF1expression, without noticeable changes in SIRT1 expressions. Consistent with this observation, overexpression Gαq-TG study suggests that Gαq is sufficient to mediate cardiac hypertrophy through AT1R activation [23]. Further, TG-HD rats showing amplified activation of IGF-IIR expression through MAPK/HSF1/SIRT1 pathway. Thus, we propose that IGF-IIRα might also activate other signaling mechanisms to regulate IGF-IIR expressions causing cardiac hypertrophy. In addition, activated IGF-IIR protein translocates to the membrane and binds to IGF-II (IGF-IIR:IGF-II) leading to Gαq mediated calcium signaling [29]. Since IGF-IIRα lacks transmembrane domain the activated protein might reside in the cytoplasm and thereby amplify hypertrophy mechanisms through regulation of IGF-IIR expressions. However, knowing its functional importance in regulating IGF-IIR expressions, future studies will evaluate its detailed mechanism of action. In converse, mechanical stress induced cardiac hypertrophy was regulated via up regulated AT1R expressions in the absence of ANG-II [30].

IGF-IIR mediate pathological hypertrophy through Gαq coupled activation of calcium/NFAT signaling. Increased calcium influx activates protein kinase C and calcineurin through phospholipase activation. Calcineurin activation hasa regulatory role during cardiac hypertrophy through NFATc3/GATA4 pathway [31–34]. NFATc3 belongs to a family of Rel homology domain-containing transcription factors [35]. NFATc3 signalling is key to transmitting hypertrophy stimuli [36–38]. In this study, calcineurin mediated dephosphorylated NFATc3 along with p-GATA4 inside the nucleus transcriptionally up-regulates ANP and BNP expressions. Increase in an active form of NFATc3 and GATA4 was found in WT-HD and TG-HD. The hypertrophic stimulus was found to be dramatically increased under high-salt conditions in TG rats. Previously, NFATc3 and GATA4 transgenic overexpression studies reveal its significant association in the development of myocardial hypertrophy [39,40]. Previous findings show that GATA4 is responsible for regulating hypertrophy and cardiac survival [41]. Calcineurin regulated NFAT/GATA4 activation causes left ventricular hypertrophy in humans [42].

## Conclusion

From these findings, it is evident that IGF-IIRα plays a crucial role in enhancing cardiac hypertrophy under high-salt conditions. Future studies on IGF-IIRα localization and specific knockdown of IGF-IIR under IGF-IIRα overexpression will delineate its exact role in cardiac hypertrophy.
